# Fructose diet alleviates acetaminophen-induced hepatotoxicity in mice

**DOI:** 10.1371/journal.pone.0182977

**Published:** 2017-08-23

**Authors:** Sungjoon Cho, Ashutosh Tripathi, George Chlipala, Stefan Green, Hyunwoo Lee, Eugene B. Chang, Hyunyoung Jeong

**Affiliations:** 1 Department of Biopharmaceutical Sciences, College of Pharmacy, University of Illinois at Chicago, Chicago, Illinois, United States of America; 2 Research Resources Center, University of Illinois at Chicago, Chicago, Illinois, United States of America; 3 Section of Gastroenterology, Knapp Center for Biomedical Discovery, University of Chicago, Chicago, Illinois, United States of America; 4 Department of Pharmacy Practice, College of Pharmacy, University of Illinois at Chicago, Chicago, Illinois, United States of America; INRA, FRANCE

## Abstract

Acetaminophen (APAP) is a commonly used analgesic and antipyretic that can cause hepatotoxicity due to production of toxic metabolites via cytochrome P450 (Cyp) 1a2 and Cyp2e1. Previous studies have shown conflicting effects of fructose (the major component in Western diet) on the susceptibility to APAP-induced hepatotoxicity. To evaluate the role of fructose-supplemented diet in modulating the extent of APAP-induced liver injury, male C57BL/6J mice were given 30% (w/v) fructose in water (or regular water) for 8 weeks, followed by oral administration of APAP. APAP-induced liver injury (determined by serum levels of liver enzymes) was decreased by two-fold in mice pretreated with fructose. Fructose-treated mice exhibited (~1.5 fold) higher basal glutathione levels and (~2 fold) lower basal (mRNA and activity) levels of Cyp1a2 and Cyp2e1, suggesting decreased bioactivation of APAP and increased detoxification of toxic metabolite in fructose-fed mice. Hepatic mRNA expression of heat shock protein 70 was also found increased in fructose-fed mice. Analysis of bacterial 16S rRNA gene amplicons from the cecal samples of vehicle groups showed that the fructose diet altered gut bacterial community, leading to increased α-diversity. The abundance of several bacterial taxa including the genus *Anaerostipes* was found to be significantly correlated with the levels of hepatic Cyp2e1, Cyp1a2 mRNA, and glutathione. Together, these results suggest that the fructose-supplemented diet decreases APAP-induced liver injury in mice, in part by reducing metabolic activation of APAP and inducing detoxification of toxic metabolites, potentially through altered composition of gut microbiota.

## Introduction

Acetaminophen (APAP) is a commonly used over-the-counter analgesic and antipyretic in the United States. Of note, an overdose (i.e., over 4 gram/day) of APAP can cause severe acute liver injury. Currently, APAP-induced hepatotoxicity is the major cause of drug-induced liver injury in the US and UK and accounts for about 56,000 emergency room visits and over 450 deaths each year [1, 2]. APAP-induced hepatotoxicity exhibits large inter-individual variability [3, 4], but it is difficult to predict or identify individuals susceptibile to the development of fulminant liver injury. Several risk factors for APAP toxicity have been reported including high dose, alcohol consumption, old age, and concomitant medications [3–5]. However, these factors explain less than half of the inter-individual variability observed in the extent of liver injury by APAP [5]. Thus, the identification of other undefined risk factors for the development of fulminant liver damage after APAP ingestion is paramount.

APAP is eliminated mainly by glucuronidation and sulfation. A small fraction (<10%) is oxidized to a highly reactive metabolite *N*-acetyl-*p*-benzoquinone imine (NAPQI) [6] through the drug-metabolizing enzymes cytochrome P450 (Cyp) 2e1 and Cyp1a2 [7–9]. The critical role of CYP-mediated APAP bioactivation in hepatotoxicity has been studied in detail using Cyp-null mice. *Cyp2e1*-null mice exhibited a higher survival rate as compared to wild-type mice; however, they were still susceptible to hepatotoxicity at high APAP doses [10]. *Cyp2e1* and *Cyp1a2* double-knockout mice have shown >90% survival after receiving APAP at doses up to 1200 mg/kg, suggesting that both CYP1A2 and CYP2E1 play major roles in APAP-induced hepatotoxicity [11]. NAPQI, which triggers hepatotoxicity by covalently modifying the thiol groups of cellular proteins [12], is detoxified by reduced glutathione (GSH) that conjugates the toxic metabolite. However, during APAP overdose, excess NAPQI depletes GSH and causes oxidative damage characterized by the production of reactive oxygen species, ultimately leading to apoptosis and hepatocellular necrosis [13]. Pretreatment of mice with GSH-depleting agents or fasting (that reduces endogenous GSH levels) potentiates APAP-induced hepatic necrosis whereas GSH precursors alleviates the severity of APAP hepatotoxicity [14, 15].

GSH is a tripeptide consisting of cysteine, glutamate, and glycine [16]. Glutamate cysteine ligase (GCL) catalyzes the synthesis of γ-glutamylcysteine (from cysteine and glutamate), the rate-limiting step in glutathione biosynthesis [17]. GCL is composed of two subunits, i.e., catalytic (GCLC) and modulatory (GCLM) subunit; GCLC is responsible for catalytic action of the enzyme, and GCLM modulates the catalytic activity of GCL by improving its binding affinity to glutamate [18, 19]. Transgenic mice that overexpress both GCLC and GCLM are protected against APAP-induced liver injury [19]. On the other hand, Gclm-null mice have shown higher susceptibility to APAP toxicity compared to wild-type mice [20], indicating the importance of GCL in modulating APAP hepatotoxicity.

Fructose is the major component of the Western diet rich in highly refined carbohydrates. In rat liver slices, fructose has shown to prevent APAP injury, potentially via generating ATP [21]. Also, rats fed with 25% (w/v) fructose diet for 5 weeks showed resistance against APAP-induced hepatotoxicity [22]. In another study, however, increased APAP hepatotoxicity was reported in rats co-administered with fructose [23]. Thus, it remains unclear whether and how fructose-rich diets alter susceptibility to APAP hepatotoxicity. The results from our study show that long-term administration of fructose decreases APAP-induced hepatotoxicity in mice, potentially by reduced bioactivation of APAP and increased detoxification of reactive metabolites.

## Materials and methods

### Reagents

APAP, APAP-d4, APAP glucuronide, APAP sulfate, 2-benzoxazolinone, chlorzoxazone, ethoxyresorufin, and protease type XIV enzyme were purchased from Sigma-Aldrich (St. Louis, MO). APAP-d3 glucuronide and APAP-d3 sulfate were from Santa Cruz Biotechnology (Dallas, TX). APAP-cysteine was purchased from Toronto Research Chemicals (North Rock, Canada). 6-Hydroxychlorzoxazone and resorufin were purchased from Cayman chemicals (Ann Arbor, MI) and Life Technologies (Eugene, OR), respectively.

### Animals

Male C57BL/6 mice (8 weeks old) purchased from Jackson Laboratory (Bar Harbor, ME) were divided into two groups (n = 20-24/group). One group was given regular water while the other group was given 30% (w/v) fructose in water for 8 weeks. Mice were monitored daily for food and water intake. After 8 weeks, the mice were subdivided into two groups (n = 9-12/group); one subgroup was treated (via oral gavage) with 600 mg/kg APAP and the other subgroup with vehicle (PEG400: 2% Tween 20: ethanol (1:7:2)). Animals were sacrificed at 24 h after APAP dosing. Blood was drawn into heparinized syringes for measurement of serum alanine aminotransferase (ALT) and aspartate aminotransferase (AST) activity [13]. The liver was removed, snap frozen, and stored at -80°C for further assays. All procedures were approved by the Institutional Animal Care and Use Committee in the University of Illinois at Chicago.

### Liver histology and measurement of ALT and AST

Liver tissues were fixed with 10% neutral buffered formalin. Fixed liver tissues were embedded in paraffin. Sections were cut and stained with hematoxylin and eosin. Blood was collected by cardiac puncture 24 h after the APAP dose. Serum ALT and AST activities were measured using a Beckman Coulter AU 400.

### Measurement of hepatic triglyceride

Liver tissues (100–120 mg) were homogenized in a mixture of 400 μL chloroform and 400 μL methanol solution briefly (5 strokes). To the mixture, PBS (200 μL) and ddH2O (100 μL) were added and homogenized again (5 strokes, 5 seconds each). The mixture was centrifuged (3,000 rpm) for 10 min at room temperature, and the bottom layer was transferred into Eppendorf tubes and dried under air stream. The dry lipid was dissolved in isopropanol/Triton X-100 mixture (100 μL; 95% isopropanol). Hepatic triglyceride content was then measured with a triglyceride measurement kit (Biovision, Milpitas, CA) following the manufacturer’s protocol.

### Quantitative real-time (qRT)-PCR

Total RNA was isolated from liver tissues using Trizol (Life Technologies) and used as template for the synthesis of complementary DNA using High Capacity cDNA Archive Kit (Applied Biosystems, Foster City, CA). Using the cDNA as template, qRT-PCR was performed using the following primers from Integrated DNA Technologies (California, USA): Cyp2e1 (Mm.PT.58.9617541), Cyp1a2 (Mm.PT.58.18171461), Hsp70 (Mm.PT.58.31570020.g), Gclc (Mm.PT.58.30656560), Gclm (Mm.PT.58.17443532), iNos (Mm.PT.58.43705194), Tnfα (forward 5′ CCCTCACACTCAGATCATCTTCT-3′; reverse 5′-GCTACGACGTGGGCTACAG-3′), Ahr (Mm.PT.58.11116644), Hnf1α (Mm.PT.58.33713899), ChREBP (Mm.PT.56a.33592172), Fgf21 (Mm.PT.58.29365871.g), Gapdh (Mm.PT.39a), and Gapdh (forward 5′ AGGATAAAGGACACTCCACCCAG-3′; reverse 5′-GGGAAGGAAATGAATGAACCG-3′). The fold change in mRNA levels was determined after normalizing the gene expression levels to those of Gapdh (2^-ΔΔCt^ method).

### Measurement of drug-metabolizing enzyme activities

S9 fractions were prepared as described previously [24], and activities of different drug metabolizing enzymes were measured based on preliminary results where linear increases in metabolite formation were demonstrated over time, substrate concentrations, and S9 protein contents. CYP1A2 activity was determined by spectrofluorometric method by measuring 7-ethoxyresorufin O-dealkylation reaction. Briefly, 7-ethoxyresorufin (at a final concentration of 0.5 μM) was incubated with S9 fraction (0.1 mg) in incubation mixtures containing NADPH-generating system (0.5 mM NADP+, 1 mM MgCl_2_, 0.2 U/L isocitrate dehydrogenase, and 0.5 mM isocitric acid). The reaction was stopped after 30 min by adding 200 μL ice cold solution of acetonitrile. Precipitated protein was removed by centrifugation, and metabolite (resorufin) formation was measured from supernatant fluorometrically using excitation and emission wavelengths of 530 and 580 nm, respectively.

CYP2E1 activity in S9 fractions was determined by using chlorzoxazone as a probe compound. Chlorzoxazone at a final concentration of 0.1 mM was incubated with S9 fraction (0.05 mg) in mixtures containg a NADPH-generating system. The reaction was stopped by adding 200 μL of acetonitrile containing 0.4 mM 2-benzoxazolinone (internal standard) after 15 min. Precipitated proteins were removed by centrifugation (12,000g, 4°C, 20 min), and the supernatant was analyzed using liquid chromatography tandem mass spectrometer (LC-MS/MS) in the negative ionization mode (QTrap 5500; Applied Biosystems, ON, Canada). The separation was performed on a XTerra MS C18 column (2.5 μm, 50 mm x 2.1 mm, i.d., Waters Corp., Milford, MA). The HPLC system (Agilent 1200, Santa Clara, CA) was operated isocratically at a flow rate of 0.3 mL/min. A mixture of acetonitrile (0.1% formic acid) (solvent A) and 0.1 mM ammonium formate (0.1% formic acid) (solvent B) was used as the mobile phase (A:B, 20:80). The detection and quantification of analytes were accomplished by multiple reactions monitoring with the transitions of *m/z* 183.9/119.9 for 6-hydroxychlorzoxazone, 167.9/132.1 for chlorzoxazone, and 133.9/64.9 for 2-benzoxazolinone. The compounds were eluted at 1.6, 2.1, and 5.4 min, respectively. Calibration curves were linear for the concentrations of 6-hydroxychlorzoxazone ranging from 0.05 to 10 μM.

Sulfotransferase activity in S9 fractions was determined by using APAP as a substrate. APAP at a final concentration of 0.1 mM was incubated with S9 fraction (0.1 mg) containing 1 mM 3’-phosphoadenosine 5’-phosphosulfate, 5 mM MgCl_2_ and 100 mM Tris-HCl (pH 7.4) (total volume 100 μL). After 10 min, the reaction was stopped by adding 100 μL of ice-cold acetonitrile containing APAP-d3 sulfate (internal standard, 200 ng/mL). Precipitated proteins were removed by centrifugation (12,000g, 4°C, 20 min), and the supernatant was analyzed using LC-MS/MS.

UDP-glucuronosyltransferase activity was determined by using APAP as a substrate. APAP at a final concentration of 0.25 mM was incubated with S9 fraction (0.2 mg) containing 2 mM uridine 5’-diphosphoglucuronic acid, 5 mM MgCl_2_, 25 μg/mL alamethicin, and 100 mM Tris-HCl (pH 7.4) (total volume 200 μL). After 10 min, the reaction was stopped by adding equal volume of ice-cold acetonitrile containing APAP-d3 glucuronide (internal standard, 100 ng/mL). Precipitated proteins were removed by centrifugation (12,000g, 4°C, 20 min), and the supernatant was analyzed using LC-MS/MS.

APAP-sulfate and APAP-glucuronide were measured using LC-MS/MS in the positive ionization mode. The separation was performed on a Atlantis T3 column (3 μm, 100 mm x 3 mm, i.d., Waters Corp., Milford, MA). Chromatographic separation was performed using 0.1% formic acid in water (solvent A) and 0.1% formic acid in acetonitrile (solvent B) at a flow rate of 0.4 mL/min. The gradient elution profile used is as follows: 95% A for 3 min, 95% to 10% A over 3 min, 10% A for 3 min, 10% to 95% A over 0.5 min, and 95% A for 5 min. The detection and quantification of analytes were accomplished by multiple reactions monitoring of *m/z* 232.1/152.1 for APAP sulfate, 235.1/155.1 for APAP-d3 sulfate, 328.1/152.1 for APAP glucuronide, and 331.1/155.2 for APAP-d3 glucuronide.

### Measurement of hepatic GSH and GSSG

GSH and GSSG content was determined in the liver homogenate using a commercially available kit from Dojindo molecular technologies (Rockville, MD). Briefly, liver tissues (~0.1 g) were homogenized in 5% sulfosalicylic acid solution. The homogenates were centrifuged at 8,000 *g* for 10 min (4°C). The supernatant was diluted ten-fold using ddH2O and stored at -80°C. Assay was performed using manufacturer’s instructions. A calibration curve was performed with standard GSH (0–40 μmol/L) and GSSG (0–25 μmol/L). GSH and GSSG concentrations were calculated, normalized against liver weight, and expressed as μmol/g liver and nmol/g liver, respectively.

### Measurement of serum APAP-protein adduct amount

Serum samples were filtered through Bio-spin 6 column (Bio-Rad, Hercules, CA) prewashed with 10 mM sodium acetate buffer (pH 6.5). The filtrate (50 μL) was incubated with protease type XIV enzyme in water (8 U/mL; 50 μL) at 37°C for 24 h. APAP-d4 (10 μL; 1000 ng/mL; internal standard) and ice-cold MeOH (750 μL) were added. The samples were centrifuged at 13,200 rpm for 10 min, and the supernatant (600 μL) was dried using Eppendorf Vacufuge^™^ concentrator. The evaporated residue was reconstituted in MeOH followed by centrifugation at 13,200 rpm for 10 min. The supernatants were analyzed using LC-MS/MS. The separation method for APAP-cysteine was same as the one for APAP-sulfate and APAP-glucoronide. The detection and quantification of analytes were accomplished by multiple reactions monitoring of *m/z* 270.9/139.8 for APAP-cysteine and 155.9/114.1 for APAP-d4.

### Cecal bacteria genomic DNA extraction

Cecal samples were collected at the time of sacrifice, snap-frozen, and stored at -80°C until analysis. Genomic DNA was extracted from the samples using a Tissue DNA Purification Kit, implemented on a Maxwell^®^ 16 automated extraction system (Promega). Genomic DNA quantity was assessed using a Qubit 2.0 fluorometer with the dsDNA BR Assay (Life Technologies, Grand Island, NY).

### High-throughput 16S rRNA gene amplicon sequence libraries

Bacterial community structure was characterized using high-throughput sequencing of 16S rRNA PCR amplicons. Briefly, the widely used primer set 341F and 806R, targeting the V3-V4 variable region of the 16S rRNA gene of bacteria, was used for amplification [25, 26], with slight modifications. A two-stage PCR or “targeted amplicon sequencing (TAS)” approach was performed to generate amplicon libraries, as described previously [27, 28]. In the first of the two-stage amplification procedure, the template was amplified using a pair of primers (341F and 806) containing 5′ linkers CS1 and CS2 linkers, as described previously [29]. PCR was performed in 10 μL reaction volumes using MyFi 2X PCR mastermix (Bioline, Taunton, MA), and the PCR conditions were as follows: 5 min initial denaturation at 95°C, followed by 28 cycles of: 95°C for 30 sec, 50°C for 30 sec, and 72°C for 60 sec. Subsequently, a second PCR reaction was established, with one μl of amplification product from the first stage used as input to the second reaction. The primers for the second stage amplifications were the AccessArray barcoding system primers (Fluidigm, South San Francisco, CA), containing Illumina sequencing adapters, sample-specific barcodes (10 bases, with a minimum hamming distance of 3), and CS1 and CS2 linkers [27]. PCR conditions for the second reaction were as follows: 5 min initial denaturation at 95°C, followed by 8 cycles of: 95°C for 30 sec, 60°C for 30 sec, and 72°C for 60 sec. Final PCR products were purified and equalized using SequalPrep Normalization Plate Kit (Thermo Fisher Scientific), according to the manufacturer’s instructions. Samples were pooled in equimolar ratio and quantified using a Qubit 2.0 fluorometer. Sequencing was performed on an Illumina MiSeq sequencer using standard V3 chemistry with paired-end, 300 base reads. Fluidigm sequencing primers, targeting the CS1 and CS2 linker regions, were used to initiate sequencing. Demultiplexing of reads was performed on the instrument.

The resulting forward and reverse reads were merged using PEAR [30]. Ambiguous nucleotides were trimmed from the ends and reads with internal ambiguous nucleotides were discarded. Primer sequences were identified using Smith-Watermann alignment and trimmed from the sequences. Reads that lacked either primer sequences were discarded. Sequences were then trimmed based on quality scores using a modified Mott algorithm with PHRED quality threshold of *p* = 0.01. After trimming, any sequences less than 350 bp were discarded. Chimeric sequences were identified using the USEARCH algorithm with the GreenGenes 13_8 reference sequences [31, 32]. The software package QIIME was then used to generate an Operational taxonomic unit (OTU) table and taxonomic summaries. Briefly, the resulting sequence files were merged with sample information. OTU clusters were generated in a *de novo* manner using the UCLUST algorithm with a 97% similarity threshold. Taxonomic annotations for each OTU were generated using the UCLUST algorithm and GreenGenes 13_8 reference with a minimum similarity threshold of 90%. Taxonomic and OTU abundance data were merged into a single OTU table and summaries of absolute abundances of taxa were generated for all phyla, classes, orders, families, genera, and species present in the dataset. A Bray-Curtis similarity matrix was calculated based on OTUs normalized to the total number of sequences for each sample using the software package Primer6 (Primer-E). Bacterial taxa showing an average percentage of OTUs over all samples smaller than 0.1% were excluded for statistical analysis.

### Statistical analysis

Most data were expressed as mean ± standard deviation (S.D.). Percentage of OTUs was expressed as mean ± standard error of the mean (SEM). Comparisons between the control and fructose groups were made by using the Student’s t-test or Mann-Whitney nonparametric test via R studio (version 0.99.903) or GraphPad Prism 7. For comparison of cecal microbiota between the control and fructose groups, *p*-value of Student’s t-test and false discovery rate (FDR) were calculated using R package multtest. Spearman rank correlation was performed to examine the correlation between bacterial taxa and mRNA level (of Cyp2e1, Cyp1a2, and Hsp70) or hepatic GSH level using Prism 7. *P*-value less than 0.05 was considered significant.

## Results

### Fructose increases hepatic lipid content

**Fig 1:**
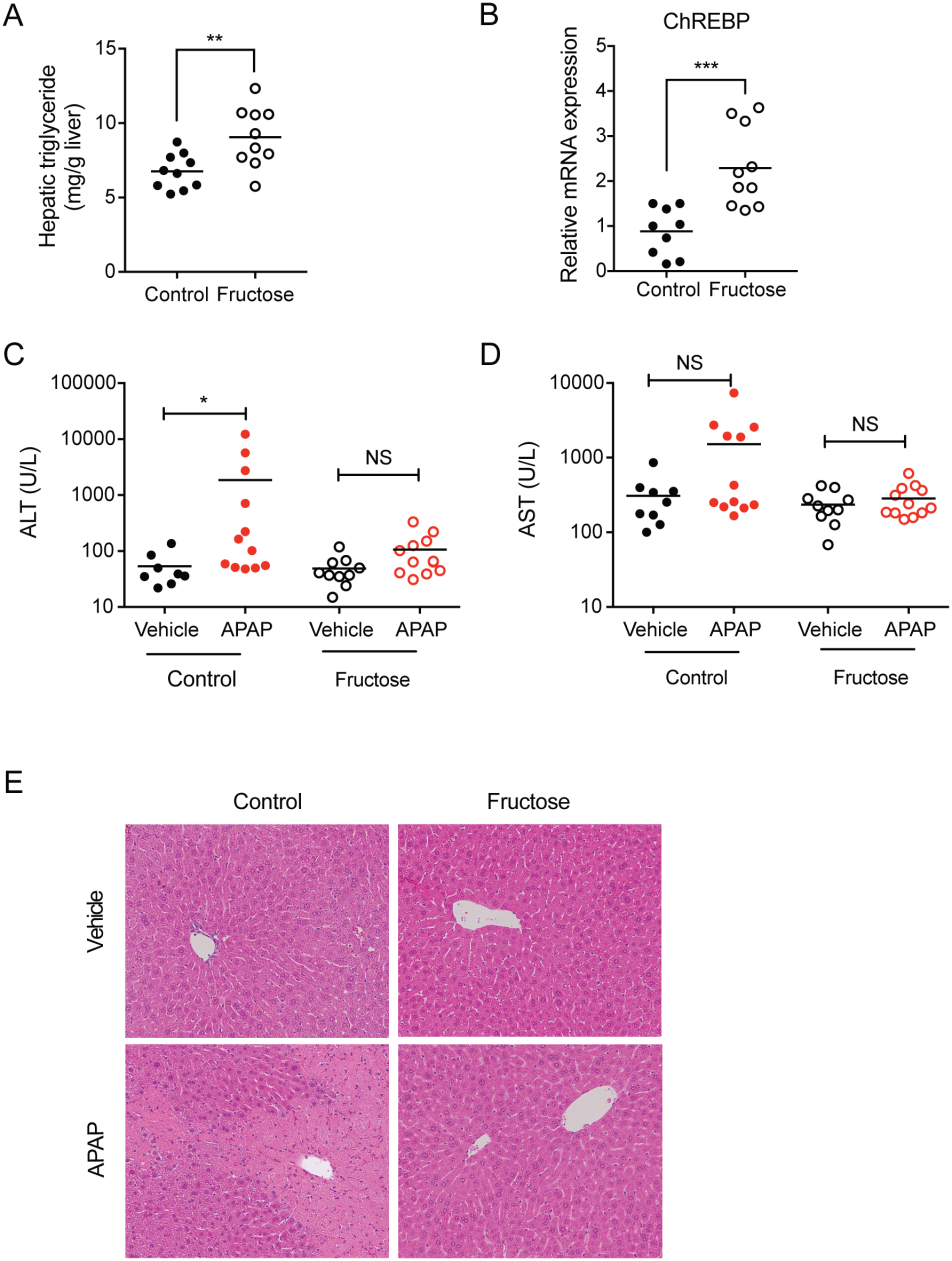
Fructose diet decreases APAP hepatoxicity. C57BL/6 mice were fed with 30% (w/v) fructose water (or control) for 8 weeks, after which APAP (600 mg/kg) or vehicle control was administered via oral gavage. Mice were sacrificed at 24 h after dosing (n = 8-12/group). (A) Liver tissues from mice administered with vehicle control were homogenized, and triglyceride content was measured. (B) Hepatic ChREBP mRNA levels were measured by qRT-PCR. (C and D) Serum ALT and AST levels in the mice were measured. (E) Liver sections were stained with hematoxylin and eosin. Representative liver sections from each group were presented (x200 maginifcation). NS; not significant; *, *p* < 0.05; **, *p* < 0.01; ***, *p* < 0.001.

Mice were fed with fructose-containing water for 8 weeks, after which APAP (or vehicle control) was administered and the extent of hepatotoxicity examined. Adding fructose to drinking water had insignificant effects on mouse body weight despite higher calorie intake (Table 1), consistent with a previous report that fructose diet did not affect body weight as it increased metabolic rate in mice [33]. Fructose is known to enhance *de novo* lipogenesis [34] in part by upregulation of carbohydrate-responsive element-binding protein (ChREBP) [35]. To further validate the animal model of fructose feeding, hepatic triglyceride level was measured. The triglyceride level as well as ChREBP expression was significantly higher in mice treated with fructose compared to control mice (Fig 1A and 1B). Together, in our experimental setting, the key features of previously reported physiological changes in fructose-fed rodents were recapitulated.

**Table 1:** Effects of fructose diet on body weight and calorie intake (n = 18-20/group).

	Control	Fructose
Body weight at the time of drug dosing (g)	29.1 ± 0.4	30.2 ± 0.4
Food intake (g/day/mouse)	4.79 ± 0.88	4.98 ± 1.16
Water intake (mL/day/mouse)	4.49 ± 0.57	6.37 ± 1.13 ***
Calorie intake (kcal/day/mouse)	14.7 ± 2.7	23.1 ± 3.7 ***

***, *p* < 0.001 vs. control

To determine the effects of fructose diet on APAP hepatotoxicity, APAP (or vehicle) was administered to the mice fed with fructose or control water for 8 weeks, and the mice were sacrificed 24 h post-dosing, the time point when APAP-induced liver damage (as determined by serum ALT levels) is greatest [36, 37]. In mice supplied with regular water, APAP significantly increased serum ALT value as expected (54 vs. 1,848 U/L for vehicle and APAP groups, respectively; Fig 1C). On the other hand, mice treated with fructose water were resistant to APAP toxicity (49 vs. 106 U/L for vehicle and APAP groups, respectively; Fig 1C). The similar trend was observed in AST values although the difference in the control group did not reach a statistical significance due to large variability.

#### Fructose diet reduces APAP bioactivation and increases detoxification

**Fig 2:**
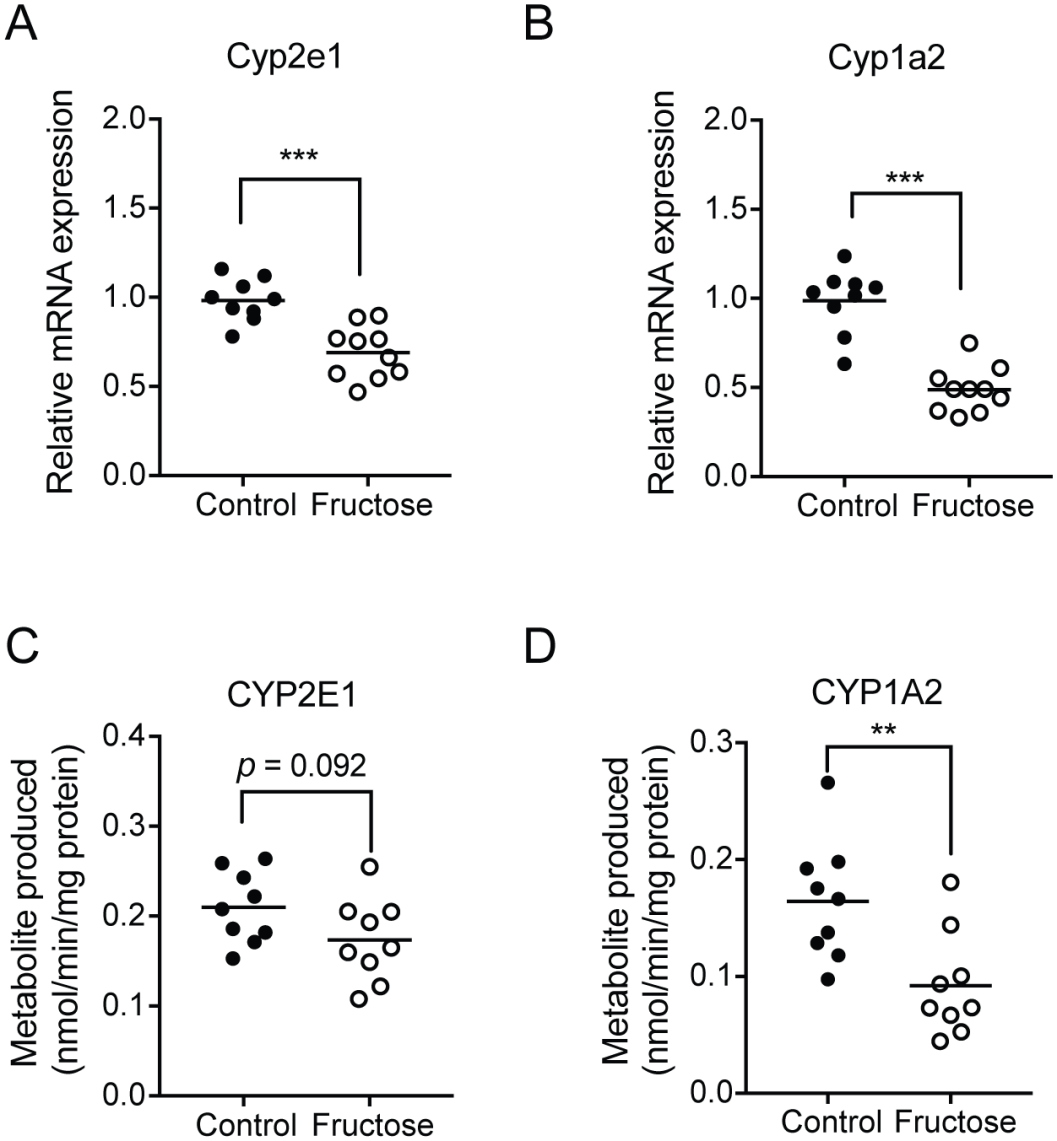
APAP bioactivation is decreased in fructose-fed mice. Mice were fed with fructose (or control) water for 8 weeks, after which vehicle was administered (via oral gavage). Mice were sacrificed at 24 h after dosing (n = 9-10/group). (A and B) mRNA expression of Cyp1a2 (A) and Cyp2e1 (B) in mouse liver tissues were measured by qRT-PCR. (C and D) The rates of ethoxyresoryufin O-deethylation (CYP1A2 activity, C) and chlorzoxazone 6-hydroxylation (CYP2E1 activity, D) were measured in hepatic S9 fractions. **, *p* < 0.01; ***, *p*< 0.001.

To determine the underlying mechanisms for decreased APAP toxicity in fructose-fed mice, we examined whether fructose alters bioactivation of APAP. Considering the fact that CYP2E1 and CYP1A2 are largely responsible for conversion of APAP into the toxic metabolite NAPQI, mRNA levels of Cyp2e1 and Cyp1a2 in the liver of mice administered with vehicle were measured by using qRT-PCR. Additionally, enzyme activities in S9 fractions were measured by using the probe drugs (i.e., 7-ethoxyresorufin and chlorzoxazone for CYP1A2 and CYP2E1, respectively). The expression of Cyp1a2 and Cyp2e1 mRNAs was significantly reduced in fructose-fed mice compared to control mice (Fig 2A and 2B). Consistently, the enzyme assays showed decreased CYP1A2 and CYP2E1 activities in fructose-fed mice (Fig 2C and 2D). The difference in CYP2E1 activities between the groups did not reach a statistical significance (Fig 2C), likely due to a larger experimental variability in measuring enzyme activities compared to measurements of mRNA levels. To further verify decreased APAP bioactivation upon fructose diet, the serum concentrations of APAP-protein adducts were measured. The concentrations of APAP-protein adducts were low, likely due to long (24 h) time lapsed since the APAP dose (S1 Fig); they were higher in the control group although the difference between the groups did not reach a statistical significance. To examine potential effects of fructose diet on APAP elimination (and the amount of APAP available for bioactivation), basal activity levels of major APAP metabolizing enzymes (i.e., sulfotransferase and UDP glucuronosyltransferase) were examined in the livers of vehicle-treated mice. Sulfotransferase activities in S9 fractions were similar between control and fructose groups (S2 Fig). Interestingly, however, basal UGT activities were higher in the control group (S2 Fig) although this did not appear to lessen APAP bioactivation in the liver, consistent with the previous report where enhanced glucuronidation of APAP (by co-treatment with rifampin) did not alter the extent of APAP bioactivation [38]. Together, our results suggest that the reduction of APAP toxicity in fructose-fed mice is due to decreased APAP bioactivation to NAPQI.

**Fig 3:**
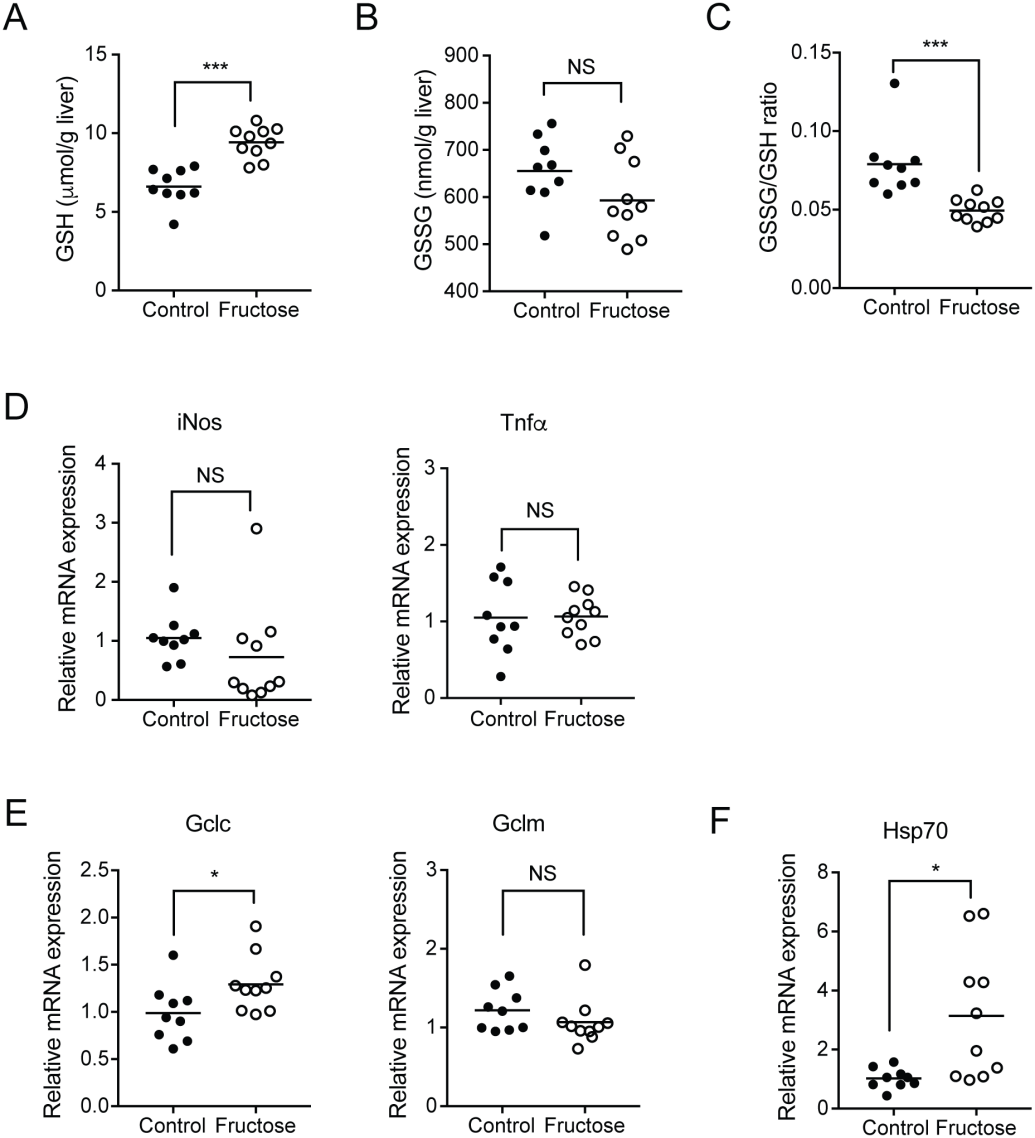
Fructose diet induces the basal hepatic glutathione content. Mice were fed with fructose (or control) water for 8 weeks, after which the mice were treated with vehicle (via oral gavage). Mice were sacrificed at 24 h after dosing (n = 9-10/group). (A and B) The contents of reduced (GSH, A) and oxidized glutathione (GSSG, B) in mouse liver tissues were measured. (C) GSSG:GSH ratio was calculated based on GSH and GSSG contents. (D-F) mRNA levels of genes in the liver tissues were measured by using qRT-PCR. NS, not significant; *, *p* < 0.05; ***, *p*< 0.001.

NAPQI is detoxified by glutathione, during which reduced glutathione (GSH) is converted to the oxidized form (GSSG) [39]. To examine whether the fructose diet alters the extent of NAPQI detoxification, the hepatic amounts of reduced and oxidized glutathione were measured in vehicle-treated mice. The results showed that basal GSH levels were increased in the fructose-fed group compared to the controls (9.42 vs. 6.61 μmol/g liver; Fig 3A). GSSG levels did not differ between the groups (Fig 3B), suggesting that the degree of basal oxidative stress is similar between the groups. This led to decreased ratios of GSSG/GSH in the fructose group (Fig 3C). Consistent with the notion of similar levels of basal oxidative stress (and subsequent inflammation), mRNA expression of inflammation markers, namely iNos (inducible nitric oxide synthase) and Tnf (tumor necrosis factor) α, did not differ between the groups (Fig 3D). Together, these results indicate that increased basal glutathione levels may in part explain the reduced APAP toxicity in fructose-fed mice.

GCL is a rate-limiting enzyme in the production of glutathione [40]. To identify potential mechanisms for increased basal glutathione levels in fructose-fed mice, mRNA expression levels of Gclc and Gclm were examined in the liver tissues of vehicle-treated mice. The mRNA level of Gclc was higher in the fructose-fed mice (Fig 3E, left), but Gclm expression did not differ between the groups (Fig 3E, right).

In addition to GSH, other cellular components including heat shock protein (HSP) dampen cellular damage by NAPQI. HSP70 was previously shown to confer protection against APAP-induced hepatotoxicity in mice [41]. Results from qRT-PCR showed that mRNA expression of Hsp70 was significantly higher in the fructose-fed group compared to the control group (Fig 3F), suggesting potential roles of Hsp70 in fructose diet-induced alleviation of APAP hepatotoxicity. Also, ChREBP target gene fibroblast growth factor (Fgf) 21 is known to confer protection against APAP-induced hepatotoxicity [42, 43], but its expression did not differ between the groups (S3 Fig).

#### Fructose diet alters gut microbiota structure

**Fig 4:**
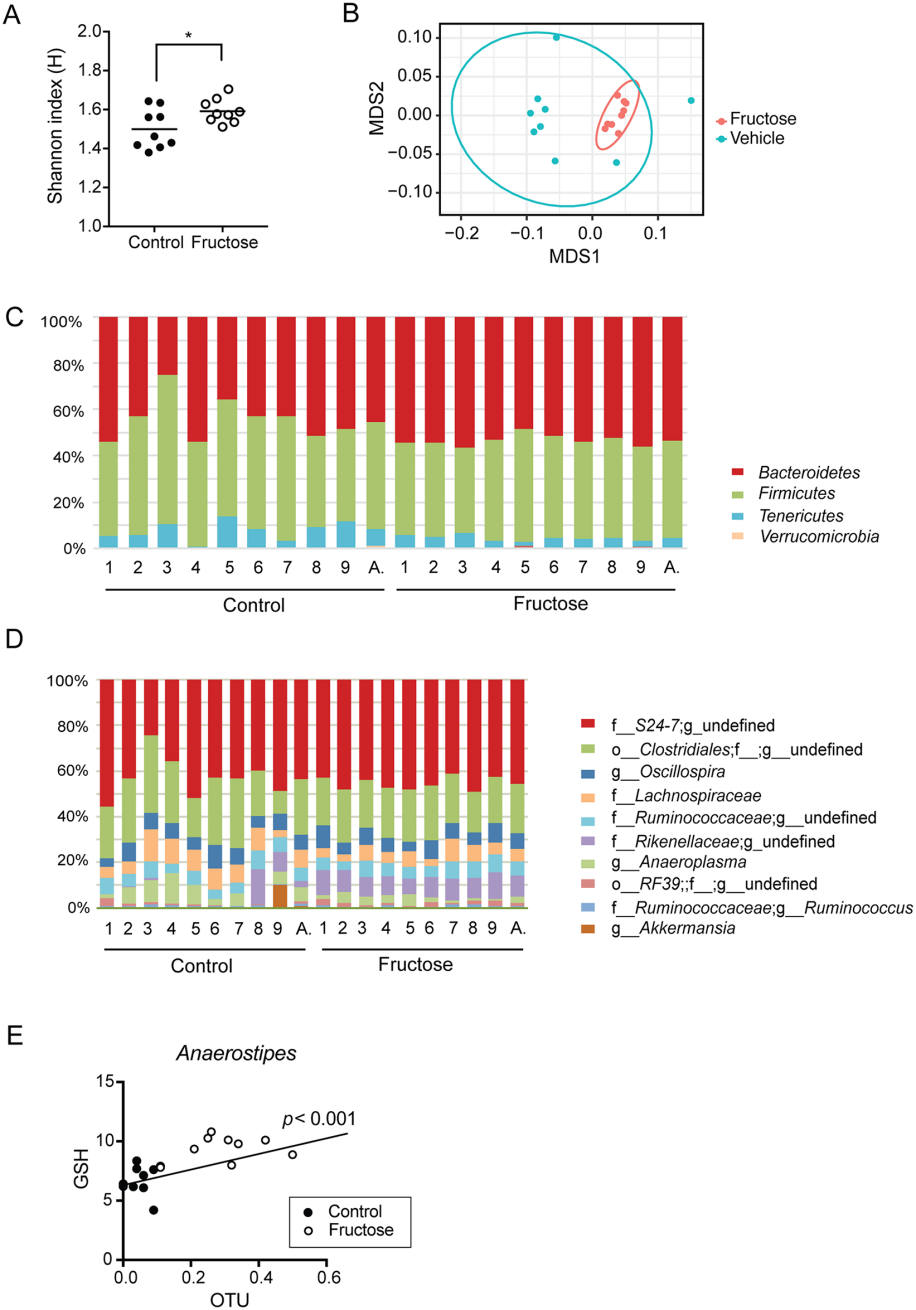
Fructose diet alters gut microbiota composition. Mice were fed with regular (control) or fructose water for 8 weeks, and then administered with the vehicle control via oral gavage (n = 9/group). Mice were sacrificed at 24 h after dosing, and cecal samples were collected. The composition of the gut microbiota was analyzed by 16S rRNA gene profiling. (A) Shannon index was calculated to compare α-diversity of each sample. (B) NMDS plot of gut microbiome from the control and fructose groups is shown. Each point represents a mouse. (C and D) Relative abundance of bacterial taxa at the phylum (C) and family (D) level. The top 10 most abundant families are shown. “A.” bar represents the average of 9 mice for each group. (E) Correlation between *Anaerostipes* OTU and hepatic GSH levels in vehicle-treated mice fed with fructose or regular water (n = 18).

Previous studies in rats have shown that fructose diet alters hepatic functions in part via modulating the composition of gut microbiota [44, 45]. To determine gut microbiota composition in mice fed with fructose diet, cecal samples were obtained from the fructose-fed and control groups. The results showed that fructose diet increased α-diversity (i.e. Shannon index) of gut microbiota (Fig 4A). Nonmetric multidimensional scaling (NMDS) plot of β-diversity (i.e. Bray-Curtis index) showed that gut microbiota from the control and fructose groups formed separate clusters (ANOSIM statistics *R* = 0.545, *p* = 0.001) (Fig 4B), indicating the presence of distinct gut microbiota community in fructose-fed mice. *Firmicutes* and *Bacteroidetes* were two dominant phyla in both control and fructose-fed groups, together accounting for over 90% of the identified bacterial taxa (Fig 4C). *Firmicutes* phyla were composed predominantly of bacteria in the *Clostridia* class (of which *Clostridiales* order constitutes 99%), and bacteria in *Bacteroidia* class were mainly from *S24-7* and *Rikenellaceae* family (Fig 4D), consistent with the previous report in C57BL/J mice [46].

Further comparison of gut microbiota between the control and fructose groups revealed that the fructose diet increased the abundance of *Rikenellaceae* family, *Bifidobacterium* genus, *Anaerostipes* genus, and *Anaeroplasma* genus (Table 2); however, only the difference in the relative abundance of *Anaerostipes* genus was statistically significant after the correction for multiple testing (i.e., FDR).

**Table 2:** Comparison of cecal bacteria in mice fed with control or fructose water (n = 9/group).

Phylum	Class	Order	Family	Genus	Conrol group*	Fructose group*	t-test *p* value	FDR
*Actinobacteria*	*Actinobacteria*	*Actinobacteria*	*Bifidobacteriaceae*	*Bifidobacterium*	0.00875 ± 0.00353	0.24 ± 0.08	**0.00901**	0.230
	*Coriobacteriia*	*Coriobacteriales*	*Coriobacteriaceae*	*Undefined*	0.0636 ± 0.0320	0.146 ± 0.057	0.225	1
*Bacteroidetes*	*Bacteroidia*	*Bacteroidales*	*Rikenellaceae*	*Undefined*	2.79 ± 1.75	9.07 ± 0.43	**0.00309**	0.118
			*S24-7*	*Undefined*	42.2 ± 3.0	44.3 ± 1.0	0.513	1
*Firmicutes*	*Bacilli*	*Lactobacillales*	*Lactobacillaceae*	*Lactobacillus*	0.0816 ± 0.0220	0.221 ± 0.073	0.0866	0.947
		*Turicibacterales*	*Turicibacteraceae*	*Turicibacter*	0.113 ± 0.037	0.107 ± 0.036	0.904	1
	*Clostridia*	*Clostridiales*	*Clostridiaceae*	*Undefined*	0.129 ± 0.048	0.337 ± 0.113	0.109	1
			*Dehalobacteriaceae*	*Dehalobacterium*	0.179 ± 0.031	0.134 ± 0.012	0.194	1
			*Lachnospiraceae*	*Anaerostipes*	0.0525 ± 0.0134	0.303 ± 0.038	**<0.0001**	**0.00107**
				*Coprococcus*	0.485 ± 0.122	0.483± 0.120	0.993	1
				*Ruminococcus*	0.117 ± 0.015	0.150 ± 0.061	0.603	1
				*Undefined*	8.16 ± 1.12	5.49 ± 0.70	0.0606	0.928
			*Ruminococcaceae*	*Oscillospira*	6.77 ± 0.62	6.67 ± 0.60	0.909	1
				*Ruminococcus*	0.912 ± 0.109	0.935 ± 0.155	0.907	1
				*Undefined*	5.89 ± 0.50	5.92 ± 0.46	0.957	1
			*Undefined*	*Undefined*	24.0 ± 2.5	21.0 ± 0.7	0.271	1
	*Erysipelotrichi*	*Erysipelotrichales*	*Erysipelotrichaceae*	*Allobaculum*	0.0119 ± 0.0089	0.316 ± 0.163	0.0809	0.947
				*Undefined*	0.111 ± 0.040	0.127 ± 0.026	0.754	1
*Tenericutes*	*Mollicutes*	*Anaeroplasmatales*	*Anaeroplasmataceae*	*Anaeroplasma*	5.94 ± 1.38	2.61 ± 0.53	**0.0381**	0.730
		*RF39*	*Undefined*	*Undefined*	0.891 ± 0.316	1.42 ± 0.26	0.212	1
*Verrucomicrobia*	*Verrucomicrobiae*	*Verrucomicrobiales*	*Verrucomicrobiaceae*	*Akkermansia*	1.10 ± 1.10	0.00556 ± 0.00159	0.325	1

* Percentage of OTUs expressed as mean ± SEM.

Bold; *p* < 0.05 between control and fructose groups.

To determine whether the differences in Cyp2e1/Cyp1a2/Hsp70 expression or GSH levels between control and fructose groups are attributable to differential gut microbiota, their correlation with individual bacteria was examined. Several bacterial taxa were found to show statistically significant correlation with GSH levels or mRNA levels of Cyp2e1, Cyp1a2, and Hsp70 (S1 Table). However, within each group of control or fructose-fed mice, the correlation was statistically insignificant (Fig 4E as an example).

## Discussion

APAP-induced hepatotoxicity is a major contributor to acute liver failure, but risk factors leading to serious liver injury after APAP ingestion remain unclear. Previous studies have reported conflicting effects of fructose, a major component in Western diet, on the susceptibility to APAP hepatotoxicity. In this study, we investigated whether and how dietary fructose modulates APAP hepatotoxicity using a mouse model.

Our results showed that a fructose-supplemented diet alleviates APAP hepatotoxicity in mice. This is accompanied by decreased expression of Cyp2e1 and Cyp1a2 and increased basal levels of GSH in the liver, likely leading to decreased bioactivation of APAP and increased detoxification of NAPQI. Overall, our finding is in line with previous reports where fructose prevented the development of hepatotoxicity during the early-stage (within 24 h after APAP challenge) [21].

The underlying mechanisms for altered heaptic Cyp1a2/Cyp2e1 expression or GSH levels are unclear. Transcription factors that are known to modulate basal expression levels of Cyp1a2 and Cyp2e1, namely aromatic hydrocarbon receptor (Ahr) [47] and hepatocyte nuclear factor (Hnf) 1α [48], did not differ (at mRNA levels) between the fructose-fed and control groups (S4 Fig), suggesting that they do not contribute to the decreased Cyp1a2 and Cyp2e1 expression in fructose-fed mice. Interestingly, hepatic mRNA levels of Cyp1a2 and Cyp2e1 as well as hepatic GSH levels are all known to be modulated by gut microbiota [49, 50]. For example, *de novo* GSH synthesis is decreased in conventionally raised mice as compared to germ-free mice due to multiple effects of gut bacteria on GSH biosynthesis. This includes the consumption of glycine by intestinal bacteria (making it less available for GSH synthesis) and the lower expression of GCL [49]. Similarly, Cyp1a2 and Cyp2e1 exhibit lower hepatic mRNA expression levels in conventionally raised mice by unknown mechanisms [50].

Currently available evidence suggests that fructose-rich diets alter hepatic function in part by modulating gut microbiota. For example, increased hepatic lipid accumulation with fructose diets was markedly reduced in mice concomitantly treated with antibiotics [51]. Despite this evidence, it remains unknown to date what structural and functional changes in gut microbiota are triggered by fructose-rich diets in mice. Our study in mice showed that the fructose diet tends to increase the abundance of bacteria in the *Rikenellaceae* family, and *Bifidobacterium* or *Anaerostipes* genus while decreasing the genus *Anaeroplasma*. After correction for multiple testing, only the differences in *Anaerostipes* remained statistically significant. The increased abundance of *Bifidobacterium* and *Anaerostipes* is in line with a previous report of their capability to metabolize fructose [52–54], in part supporting the validity of our finding. Other biological roles of *Anaerostipes* are largely unknown. In assessing potential correlations between gut microbiome and hepatic levels of Cyp1a2, Cyp2e1, Hsp70, and GSH, we found a statistically significant association for certain bacterial taxa (including *Anaertostipes*). However, such association was not observed within each (control or fructose) group. While this suggests that the correlation between bacterial taxa and differential Cyp1a1/Cyp2e1 expression or GSH levels may be driven mainly by the differences in gut microbiota composition between the fructose and control diet groups (rather than the effects of specific bacterial taxa on hepatic function), the sample size used in our study (i.e., n = 9/group) was likely too small to detect such correlation especially if it is weak. Potential cause-and-effect relationships between the changes in Cyp1a2/Cyp2e1 expression (or GSH level) and gut microbiota remain to be further investigated.

Our results showing decreased APAP hepatotoxicity with fructose-rich diets are consistent with previous reports in rats [22], but the opposite finding (i.e., fructose increasing the severity of APAP hepatotoxicity in rats) has also been reported [23]. The liver is continuously exposed to bacterial cell surface products from the small intestine such as the lipopolysaccharide (LPS) that can cause low-grade hepatic inflammation [55]. In a recent study by Schirmer et al, variations in inflammatory response (i.e., production of TNFα from peripheral blood mononuclear cells upon LPS challenge) in 500 healthy individuals is partly ascribed to differences in individual gut microbiota [56], suggesting a potential role of differential gut microbiota in determining the extent of hepatic inflammation. Interestingly, molecules such as LPS that enhance hepatic inflammation are known to worsen APAP-induced hepatotoxicity [57, 58], suggesting that different levels of basal inflammation in the liver may explain the discrepancy in the effects of the fructose diet on APAP toxicity. Indeed, the expression of inflammatory marker genes (e.g., iNos and Tnfα) did not differ between the control and fructose groups in this study, suggesting that fructose diet did not cause inflammation in our experimental setting unlike in a previous study [51]. Taken together, our and other results suggest the need to examine the changes in gut microbiota as a potential contributor to altered liver physiology as well as the basal inflammation levels as a potential confounding factor.

Our study showed that the fructose diet increased the basal expression of Hsp70. The expression of HSPs is known to be highly upregulated upon cellular stress, conferring resistance to a variety of cytotoxic insults [59]. The hepatic expression of Hsp70 is known to increase after APAP dose, likely responding to the formation of protein adducts incurred by APAP [60]. On the other hand, it is rather unclear whether a basal expression level of Hsp70 also determines the susceptibility of APAP hepatotoxicity. Conflicting results have been reported in studies using *Hsp70*-null mice: increased serum ALT levels in *Hsp70*-null mice at 24 h post-dosing (vs. the wild-type mice) in one study [41] and similar ALT levels between the wild-type and *Hsp70*-null mice in another study [61]. This discrepancy may also be due to different levels of basal hepatic inflammation and suggests that Hsp70 plays a relatively minor role in protecting against APAP-induced hepatotoxicity. Therefore, it appears unlikely that the increased Hsp70 confers protection to APAP hepatotoxicity in fructose-fed mice.

Taken together, our study demonstrates that fructose feeding modulates the susceptibility to APAP-hepatotoxicity, a finding associated with changes in the community structure of the gut microbiota. Our results showing a correlation between the abundance of certain bacterial taxa and the hepatic levels of Cyp1a2/Cyp2e1 (as well as GSH) underscores the possibility that the changes in gut microbiota structure (upon fructose diet) may modulate susceptibility to acetaminophen hepatotoxicity and provides a basis to investigate the potential roles of specific gut microbes in modulating the susceptibility to APAP-induced hepatotoxicity.

## Supporting information

S1 Table Correlation between bacterial taxa and hepatic gene expression or GSH level. (PDF)

S1 Fig Serum concents of APAP-protein adduct in APAP-treated mice. (PDF)

S2 Fig Basal activities of sulfotransferase and UDP glucuronosyltransferase. (PDF)

S3 Fig Basal Fgf21 expression in mouse livers. (PDF)

S4 Fig Basal AhR and Hnf1α expression in mouse livers. (PDF)

## Abbreviations

Ahr: aromatic hydrocarbon receptor
ALT: alanine aminotransferase
APAP: acetaminophen
APAP-cys: acetaminophen-cysteine
AST: aspartate aminotransferase
ChREBP: carbohydrate-responsive element-binding protein
Cyp: cytochrome P450
FDR: false discovery rate
Fgf21: fibroblast growth factor 21
GCLC: glutamate cysteine ligase, catalytic domain
GCLM: glutamate cysteine ligase, modulatory domain
GSH: reduced glutathione
Hnf1α: hepatocyte nuclear factor (Hnf) 1α
Hsp: heat shock protein
iNos: inducible nitric dxide synthase
LC-MS/MS: liquid chromatography tandem mass spectrometer
LPS: lipopolysaccharide
NAPQI: *N*-acetyl-*p*-benzoquinone imine
OTUs: operational taxonomic units
Tnfα: tissue necrosis factorα
