# Supplementary material for: Fructose diet alleviates acetaminophen-induced hepatotoxicity in mice
Source: PLoS One. 2017 Aug 23;12(8):e0182977. doi: 10.1371/journal.pone.0182977 (PMC5568217; doi:10.1371/journal.pone.0182977)
Supplement: S1 Table — (PDF) [file pone.0182977.s001.pdf]

S1 Table. Correlation between bacterial taxa and hepatic gene expression or GSH levels

Dependent variable	Bacterial taxa	Spearman coefficient r	*p*-value
CYP1A2 mRNA level	*g_Bifidobacterium*	-0.813	<0.001
	f_Rikenellaceae;g_undefined	-0.522	<0.05
	g_Lactobacillus	-0.550	<0.05
	f_ Clostridiaceae;g_undefined	-0.490	<0.05
	g_Anaerostipes	-0.710	<0.001
	g_Akkermansia	0.565	<0.05
CYP2E1 mRNA level	*g_Bifidobacterium*	-0.767	<0.001
	f_Rikenellaceae;g_undefined	-0.596	<0.01
	g_Anaerostipes	-0.583	<0.05
	g_ Akkermansia	0.513	<0.05
Hsp70 mRNA level	*g_Anaerostipes*	0.542	<0.05
	g_Akkermansia	-0.757	<0.01
GSH level	*g_Bifidobacterium*	0.680	<0.01
	f_Rikenellaceae;g_undefined	0.593	<0.01
	f_Clostridiaceae;g_undefined	0.497	<0.05
	g_Anaerostipes	0.785	<0.001
	g_Akkermansia	-0.728	<0.001
