# Supplementary material for: Fructose diet alleviates acetaminophen-induced hepatotoxicity in mice
Source: PLoS One. 2017 Aug 23;12(8):e0182977. doi: 10.1371/journal.pone.0182977 (PMC5568217; doi:10.1371/journal.pone.0182977)
Supplement: S1 Fig — (PDF) [file pone.0182977.s002.pdf]

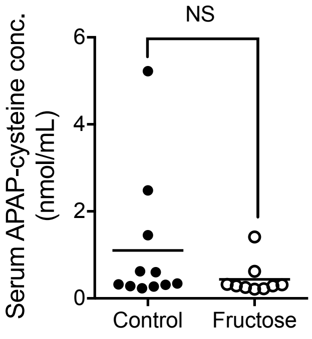

S1 Fig. Serum concentrations of APAP-protein adduct in APAP-treated mice. Mice were fed with fructose (or control) water for 8 weeks, after which vehicle APAP was administered via oral gavage. Mice were sacrificed at 24 h after dosing (n=9-10/group). The contents of APAP-protein adduct in serum of APAP-treated mice were estimated by measuring APAP-cysteine after size exclusion chromatography followed by protein digestion. NS; not significant.
