# Supplementary material for: Fructose diet alleviates acetaminophen-induced hepatotoxicity in mice
Source: PLoS One. 2017 Aug 23;12(8):e0182977. doi: 10.1371/journal.pone.0182977 (PMC5568217; doi:10.1371/journal.pone.0182977)
Supplement: S2 Fig — (PDF) [file pone.0182977.s003.pdf]

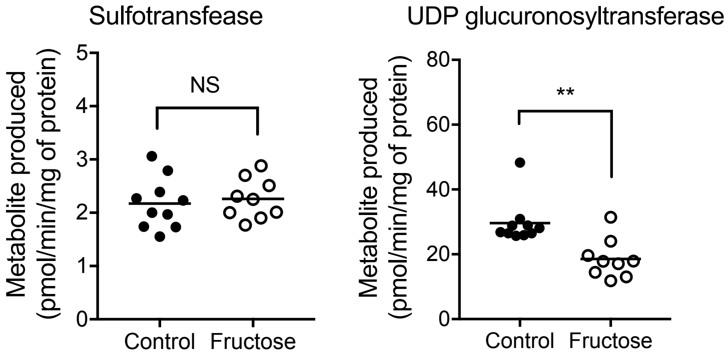

S2 Fig. Basal activities of sulfotransferase and UDP glucuronosyltransferase. Mice were fed with fructose (or control) water for 8 weeks, after which vehicle was administered (via oral gavage). Mice were sacrificed at 24 h after dosing (n=9-10/group). Activities of sulfotransferase and UDP-glucuronosyltransferase were estimated in S9 fractions from vehicle-treated mice by using APAP as a substrate. NS; not significant; **, $p < 0.01$.
