# Supplementary material for: Fructose diet alleviates acetaminophen-induced hepatotoxicity in mice
Source: PLoS One. 2017 Aug 23;12(8):e0182977. doi: 10.1371/journal.pone.0182977 (PMC5568217; doi:10.1371/journal.pone.0182977)
Supplement: S3 Fig — (PDF) [file pone.0182977.s004.pdf]

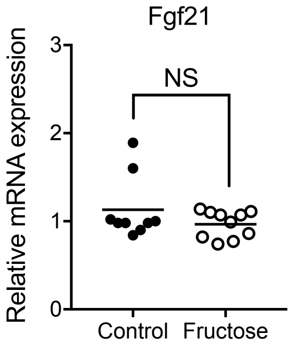

S3 Fig. Basal Fgf21 expression in mouse livers. Mice were fed with fructose (or control) water for 8 weeks, after which the mice were treated with vehicle (via oral gavage). Mice were sacrificed at 24 h after dosing (n=9-10/group). Fgf21 mRNA levels in the liver tissues were measured by using qRT-PCR. NS; not significant.
